# Blocking TIGIT/CD155 signalling reverses CD8^+^ T cell exhaustion and enhances the antitumor activity in cervical cancer

**DOI:** 10.1186/s12967-022-03480-x

**Published:** 2022-06-21

**Authors:** Lu Liu, Aihong Wang, Xiaoli Liu, Sai Han, Yu Sun, Junhua Zhang, Lingyu Guo, Youzhong Zhang

**Affiliations:** 1 Department of Obstetrics and Gynecology, Qilu Hospital, Cheeloo College of Medicine, Shandong University, Address: No.107, west culture road, Jinan, 250012 Shandong China; 2 Key Laboratory of Gynecologic Oncology of Shandong Province, Jinan, China; 3 Shandong Engineering Laboratory for Urogynecology, Qilu Hospital of Shandong University, Jinan, China; 4 Department of Obstetrics and Gynecology, Feicheng Hospital Affiliated to Shandong First Medical University, Tai’an, China

**Keywords:** Immune checkpoint, TIGIT/CD155, CD8^+^ T cells, Cervical cancer, Immunotherapy

## Abstract

**Objective:**

TIGIT/CD155 has attracted widespread attention as a new immune checkpoint and a potential target for cancer immunotherapy. In our study, we evaluated the role of TIGIT/CD155 checkpoints in the progression of cervical cancer.

**Methods:**

The expression of CD155 and TIGIT in cervical cancer tissues was detected using flow cytometry, immunohistochemistry (IHC) and gene expression profiling. In vivo and in vitro experiments have proven that blocking TIGIT/CD155 restores the ability of CD8^+^ T cells to produce cytokines. Changes in the NF-κB and ERK pathways were detected using western blotting (WB) after blocking TIGIT/CD155 signalling.

**Results:**

TIGIT expression was elevated in patients with cervical cancer. High TIGIT expression in CD8^+^ T lymphocytes from patients with cervical cancer promotes the exhaustion of CD8^+^ T lymphocytes. In addition, CD155 is expressed at high levels in cervical cancer tissues and is negatively correlated with the level of infiltrating CD8^+^ T cells. We found that TIGIT, upon binding to CD155 and being phosphorylated, inhibited NF-κB and ERK activation by recruiting SHIP-1, resulting in the downregulation of cytokine production. Blocking TIGIT in activated CD8^+^ T cells attenuates the inhibitory effect of SHIP-1 on CD8^+^ T cells and enhances the activation of NF-κB and ERK. In vivo and in vitro experiments have proven that blocking TIGIT/CD155 restores the ability of CD8^+^ T cells to produce cytokines. Injecting the blocking antibody TIGIT in vivo inhibits tumour growth and enhances CD8^+^ T lymphocyte function. Treatment with a combination of TIGIT and PD-1 inhibitors further increases the efficacy of the TIGIT blocking antibody.

**Conclusions:**

Our research shows that TIGIT/CD155 is a potential therapeutic target for cervical cancer.

**Supplementary Information:**

The online version contains supplementary material available at 10.1186/s12967-022-03480-x.

## Introduction

Cervical cancer ranks fourth in morbidity and mortality among female malignancies [1]. In 2018, 570,000 new patients were diagnosed with cervical cancer and 311,000 deaths occurred worldwide [2]. Vaccination against human papillomavirus (HPV) and cervical cancer screening effectively prevent cervical cancer [3]. Surgery is the most commonly used treatment for early cervical cancer, and it has achieved great success. Conventional treatment options for metastatic/recurrent cervical cancer include radiotherapy and chemotherapy, but this treatment exerts limited effects on advanced disease [4]. Tumour cells activate a robust immune response, but cancer still occurs. The immune response induced by tumour cells is insufficient to prevent cancer development, or a short-term immune response triggers a specific immune tolerance mechanism in tumour cells [5]. A healthy immune system requires a balance of immune checkpoint blockade (ICB). In recent years, the development of immunomodulatory drugs that block the ICB pathway has become a promising strategy for treating various malignant tumours. An anti-programmed cell death 1 (PD-1) mAb and anti-cytotoxic T lymphocyte-associated antigen 4 (CTLA-4) mAb are treatments studied in a variety of malignant tumours, including cervical cancer [6, 7]. However, the therapeutic effects of the anti-PD-L1 mAb and anti-PD-1 mAb are limited. Only 14% and 26.3% of patients achieve a complete response (CR) or partial response (PR) to pembrolizumab and nivolumab during treatment of cervical cancer, respectively [8, 9]. Therefore, we must find immunomodulators targeting other immune checkpoints to treat malignant tumours.

Yu et al. discovered TIGIT, a gene expressed on T and natural killer (NK) cells that encodes a protein with a variable immunoglobulin domain and an immunoglobulin tail tyrosine (ITT)-like phosphorylation motif followed by an ITIM (immunoreceptor tyrosine-based inhibition motif) of the cytoplasmic tail. TIGIT is an inhibitory receptor expressed primarily on NK cells, CD8^+^ T cells, CD4^+^ T cells, and regulatory T cells (Tregs) [10]. TIGIT may play a role in the cancer immunity cycle through its ITIM. CD155 is a high-affinity receptor for TIGIT. CD155 interacts with the ITIM domain of TIGIT, impairing T cell function. TIGIT is overexpressed in tumor-infiltrating lymphocytes (TILs) from various solid tumours, including melanoma, glioblastoma,, non-small cell lung cancer, and liver cancer [11–14] Furthermore, TIGIT is linked to poor clinical outcomes of cancer. TIGIT expression in TILs from patients with melanoma is associated with tumour metastasis [15], and TIGIT expression on CD8^+^ T cells in the PBMCs from patients with gastric cancer is associated with shorter survival [16]. TIGIT suppresses the immune system by binding to three ligands on tumour cells: CD155, CD112, and CD113. TIGIT has a high affinity for CD155 [10]. Our research results showed that TIGIT is expected to become a new target for the treatment of cervical cancer, and treatment with its blocking antibody alone or in combination with anti-PD-1/PD-L1 antibodies exerts significant effects in preclinical models of cervical cancer.

The number of CD8^+^TIGIT^+^ cells is significantly increased in patients with cervical cancer, according to our findings. Cytokine secretion by CD8^+^TIGIT^+^ cells is decreased. CD155, which is expressed in cervical cancer cells, interacts with TIGIT and impairs CD8^+^ T cell effector function. Blocking the TIGIT/CD155 pathway improves CD8^+^ T cell effector function and slows tumour progression. This study identifies a possible therapeutic target for cervical cancer. SHIP is a SH2 domain-containing inositol polyphosphate 5-phosphatase that was shown to mediate inhibition upon binding the phosphorylated ITIM. SHIP-1 is mainly expressed in hematopoietic cells and is a potent negative regulator of immune cells. SHIP-1 deficiency impairs T and B cell development. After TIGIT/CD155 ligation, ITT-like motifs are phosphorylated and SH2-containing inositol phosphatase 1 (SHIP-1) is recruited, which prematurely terminates MAPK and NF-κB signalling, leading to the downregulation of CD8^+^ T cell function.

## Materials and methods

### Patients and tissue samples

One hundred thirteen peripheral blood samples were collected, including 42 from patients with cervical cancer, 35 from patients with high-grade squamous intraepithelial lesion (HSIL), and 36 from subjects with a normal cervix. In addition, we collected 11 pairs of cervical cancer and adjacent tissues. All patients with cervical cancer included in this study underwent primary surgery. All samples were collected from the Qilu Hospital of Shandong University. The study was approved by Qilu Hospital's ethics committee.

### Cell isolation

Peripheral blood mononuclear cells (PBMCs) were isolated from whole blood using Ficoll (TBD Science, Tianjin, China) density gradient centrifugation. We obtained single cell suspensions from fresh tumour tissue. According to the manufacturer’s instructions, a gentle MACS C tube (Milteny Biotec, Bergisch Gladbach, Germany) was used for mechanical dissociation and a tumour dissociation kit (Milteny Biotec) was used for enzymatic hydrolysis. The digested cells were filtered through a 70 µm mesh, centrifuged with Ficoll (Solarbio, Beijing), and the monocytes were resuspended in RPMI 1640.

### Cell culture

The mouse cervical cancer cell line U14 was obtained from the Academy of Medical Sciences (Beijing, China). U14 cells were cultured in DMEM nmented with 10% foetal bovine serum (all from Gibco, Grand Island, NY, USA), 50 U/mL penicillin and 50 mg/mL streptomycin (all from Solarbio Science & Technology, Beijing, China). CD8^+^ T cells were purified from PBMCs through positive selection using a kit (Milteny Biotec, Bergisch Gladbach, Germany). CD8^+^ T cells were stimulated with an anti-CD3/CD28 antibody (Stemcell, Canada) in T cell expansion medium (Stemcell, Canada). Activated CD8^+^ T cells were treated with 5 μg/mL CD155-Fc (R&D Systems), or 10 μg/mL CD155-Fc. Activated CD8^+^ T cells were cocultured with tumour cells at a 10:1 ratio. Next, 10 μg/ml anti-PD-1 mAb or 5 μg/ml anti-TIGIT mAb (R&D Systems) were added to the cells. We used α-human IgG1 (R&D Systems) as an isotype control. After 48 h, CD8^+^ T cells were collected to determine cytokine production using the T cell function assay.

### Flow cytometry

PBMCs isolated from patients with cervical cancer or normal people were stained with fluorochrome-conjugated PE-conjugated-anti-CD8 (Elabscence, Wuhan, China) and PE-conjugated-anti-TIGIT-FITC (eBioscience) antibodies for 30 min at 4 °C**.** The samples were collected and flow cytometry was used for detection. For intracellular staining, cells stained with antibodies against cell surface markers were fixed and permeabilized with a fixation and permeabilization kit (BD Bioscience) for 20 min, and then treated with the fluorochrome-conjugated antibodies APC-conjugated-anti-TNF-α (eBioscience), and APC-conjugated-anti-IFN-γ (eBioscience), APC-conjugated-anti-GranzymeB (eBioscience) for intracellular staining at 4 °C for 30 min. Finally, the stained cells were analysed using a FACS Calibur flow cytometer (Becton Dickinson, USA), and the data were analysed using Flow Jo software.

### Immunohistochemistry (IHC)

For immunohistochemical analysis, the sections were deparaffinized, and then citric acid buffer was used for heat-mediated antigen retrieval. For testing, we followed the manufacturer's instructions and use an immunohistochemistry detection kit (Zhongshan Jinqiao, Beijing, China). Sections were incubated at 4 °C overnight with primary antibodies in PBS (anti-human TIGIT, 1:100, Cell Signaling Technology, Danvers, MA; anti-human CD155, 1:100, Cell Signaling Technology Danvers, MA; anti-human CD8, Abcam, Cambridge, UK; anti-mouse CD8, 1:200, Cell Signaling Technology Danvers, MA). Sections were then incubated with a biotin-labelled goat anti-rabbit IgG secondary antibody for 10 min at 37 °C. Streptavidin-conjugated peroxidase was incubated with sections for 15 min at 37 °C before staining with DAB (Zhongshan Jinqiao, Beijing, China). Meyer's haematoxylin (Solarbio Science & Technology, Beijing, China) was used to stain sections for 5 min. The slices were sealed with neutral resin after they were dehydrated.

### Multiplex immunohistochemistry (mIHC)

For the immunofluorescence analysis, we used multiple fluorescence immunohistochemical staining kits (Absin, Shanghai, China). Heat-mediated antigen retrieval and primary antibody incubation were performed using the same procedures as those described for immunohistochemistry. After an incubation with the secondary antibody for 10 min, the sections were incubated with the fluorescent staining amplification solution for 10 min at 37 °C. After three washes with TBST, sections were incubated with 4′,6-diamidino-2-phenylindole (DAPI) for 5 min. Finally, an anti-fluorescence quenching agent was used to seal the slides.

### Real-time quantitative RT-PCR (qRT-PCR)

TRIzol reagent (Invitrogen) was used to extract total RNA from cells. After total RNA was quantified using spectrophotometry, reverse transcription was performed using the PrimeScript RT kit (Accurate Biology, Hunan, China). Real-time PCR was performed using SYBR Premix Ex Taq (Accurate Biology, Hunan, China) and a 7900HT fast real-time PCR system (Applied Biosystems, Waltham, MA, USA). The primer sequences for the TIGIT, PD-1, LAG3, Tim3 and β-actin genes are shown in the Additional file 1: Table S1. The mRNA level of a specific gene was normalized to β-actin.

### Western blot

After washing the cells three times with PBS, they were lysed on ice in radioimmunoprecipitation analysis buffer (RIPA; Beyotime, China Institute of Biotechnology, 1% phenylmethylsulfonyl fluoride (PMSF), and 1% NaF for 30 min. The samples were centrifuged at 12,000 rpm for 10 min at 4 °C, and the supernatant was collected. Next, the proteins were separated on SDS–PAGE gels and transferred to a PVDF membrane (Merck Millipore, Burlington, Massachusetts, USA). The membrane was incubated with primary antibodies against β-actin (1:1000, Cell Signaling Technology), TIGIT (1:1000, Cell Signaling Technology), SHIP-1 (1:1000, Cell Signaling Technology), ERK (1:1000, Cell Signaling Technology), p-ERK (1:1000, Cell Signaling Technology), p-IκBα (1:1000, Cell Signaling Technology), p-NF-κBP65 (1:1000, Cell Signaling Technology) overnight at 4 °C, and then incubated with the appropriate secondary antibody. Image J software (National Institutes of Health) was used to analyse relative protein levels, and β-actin was used as an endogenous control.

### Immunoprecipitation

The cells were placed in lysis buffer (Beyotime Biotechnology, China), lysed on ice for 30 min, and centrifuged at 15,000 rpm for 15 min at 4 °C. Ten milligrams of antibody were incubated with 1000 mg of protein supernatant at 4 °C overnight. The supernatant was collected and incubated with Protein A/G Sepharose beads (Santa Cruz, USA) for 6 h. The beads were washed three times and boiled before the immunoprecipitated protein was detected using western blotting.

### Cas9-sgRNA knockout

Cas9 and single-guide RNA (sgRNA) lentiviruses were designed and constructed by OBiO Technology Company (Shanghai, China). A lentivirus containing Cas9 and sgRNA (sg-scramble, sg-CD155) was introduced into U14 cells. The sgRNA sequence used was ATTCGACAGGCGTCTTGGGAGGG. After 48 h, the transfected cells were selected in a medium containing puromycin. Compared with the control group, the silencing efficiency in U14 cells was approximately 99%.

### In vivo* treatments*

Female C57BL6 mice (18–22 g, 4–6 weeks old) were purchased from Beijing Vital River Laboratory Animal Technology Co., Ltd. for this study. U14 cells were trypsinized, resuspended in PBS, and 200 ml (1 × 10^7^ cells) of the cell suspension were subcutaneously injected into the right armpit of each mouse. For in vivo blockade, 3 days after the injection of the cell suspension, mice were randomly allocated to the anti-PD-1 mAb (100 μg, clone BE0188, BioXcell, West Lebanon, USA), anti-TIGIT mAb (100 μg, clone 1G9, BioXcell, West Lebanon, USA), anti-TIGIT mAb + anti-PD-1 mAb and IgG (mouse IgG1, clone MOPC-21, BioXcell) groups. Intraperitoneal injections of the blocking antibody and isotype control were administered three times a week. Mice were subcutaneously inoculated with 1 × 10^6^ U14-NC-CD155 or U14-KO-CD155 cells to investigate the antitumor effects of the target CD8^+^ T cells.

### Statistical analysis and bioinformatics analysis

GraphPad Prism 7.0 software (GraphPad Software, San Diego, CA) was used to analyse statistical significance. The results are presented as the mean ± standard deviation. The two-tailed Student’s t test was used for statistical comparisons between two independent groups; a nonparametric test was used for data that did not conform to a normal distribution. Gene expression and clinical annotation data were downloaded from GEO (Gene Expression Omnibus) and TCGA (The Cancer Genome Atlas). The “limma” package was used to analyse differentially expressed genes between cervical cancer and normal tissues. For all experiments, a p value less than 0.05 indicated a significant difference (P value are listed as follows: *P < 0.05, **P value < 0.01, ***P < 0.001).

## Results

### TIGIT was expressed at high levels in patients with cervical cancer

**Figure. 1:**
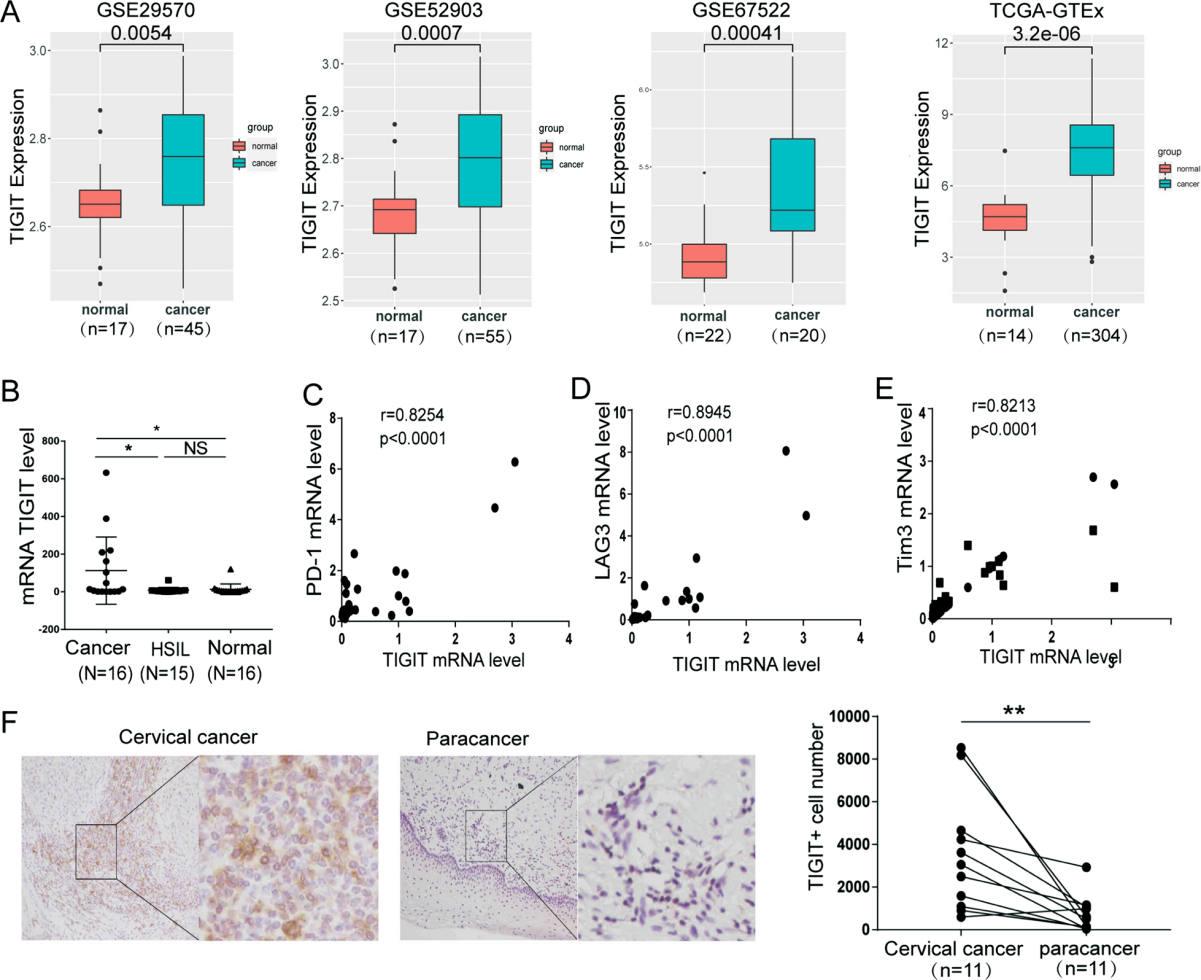
The TIGIT expression level in patients with cervical cancer. **A** Analysis of the TIGIT expression levels in cervical cancer tissues and normal cervical tissues from the GSE29570, GSE52903, GSE67522 and TCGA-GTEx databases. **B** The TIGIT expression level in PBMCs from patients with cervical cancer, patients with HSILs and individuals with normal cervix. **C**–**E** Correlation analysis of TIGIT and PD-1, TIGIT and LAG3 expression, TIGIT and Tim3 expression in PBMCs from patients with cervical cancer. **F** The level of TIGIT^+^ cells in cervical cancer tissues and adjacent tissues. The data are the mean ± SEM of the experiments. *P < 0.05, **P < 0.01

We analysed the differential expression of TIGIT in cervical cancer and normal cervical tissues from TCGA and GEO databases to determine whether TIGIT is involved in cervical cancer progression. TIGIT was expressed at significantly higher levels in patients with cervical cancer than normal people (Fig. 1A). We assessed TIGIT expression in PBMCs from 16 patients with cervical cancer, 15 patients with high-grade squamous intraepithelial lesion (HSIL), and 16 patients with normal cervix. As shown in Fig. 1B, TIGIT expression in patients with cervical cancer was significantly higher than that in patients with HSIL and normal cervix. Co-expression of immune checkpoint molecules may increase immunosuppression [17, 18]. Therefore, we tested the expression of TIGIT with coinhibitory receptor PD-1, lymphocyte activation gene 3 protein (LAG3), and T cell immunoglobulin and mucin domain-containing protein 3 (Tim3) in the PBMC of cervical cancer. We observed that TIGIT was positively correlated with PD-1, LAG3, Tim3 on CD8^+^ T cells from PBMC of patients with cervical cancer (Fig. 1C, D, E). The immunohistochemical results of 11 pairs of cervical cancer and adjacent tissues showed that the number of TIGIT positive cells in cervical cancer tissues increased significantly (Fig. 1F). The samples were shown in Additional file 2: Table S2.

### ***CD8***^+^***TIGIT***.^+^***cells was highly expressed in cervical cancer patients***

**Figure. 2:**
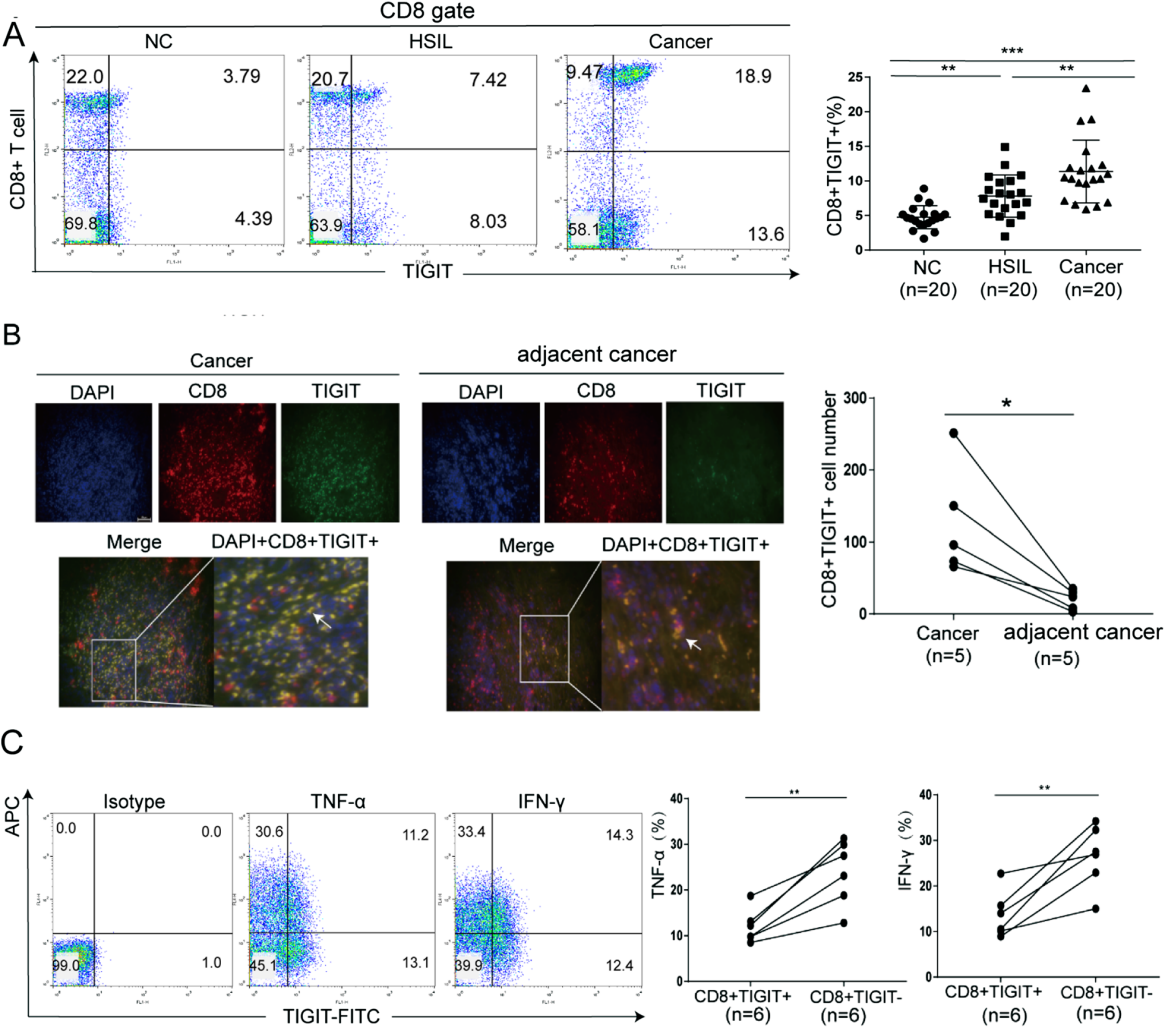
The level of CD8^+^TIGIT^+^ cells in patients with cervical cancer was related to CD8^+^ T cell depletion. **A** The proportion of CD8^+^TIGIT^+^ lymphocytes gradually increased in individuals with a normal cervix, patients with HSILs, and patients with cervical cancer. **B** The expression of TIGIT in CD8 + T cells was detected using mIHC. The level of CD8^+^TIGIT^+^ cells in cervical cancer tissues and adjacent tissues. **C** CD8^+^ T cells were isolated from PBMCs. The levels of TNF-α and IFN-γ secreted by CD8^+^TIGIT^+^ cells and CD8^+^TIGIT^−^ cells were detected by intracellular staining. The data are the mean ± SEM of the experiments. *P < 0.05, **P < 0.01

We assessed PBMCs from 20 cervical cancer patients, 20 HSIL patients, and 20 patients with normal cervix for TIGIT expression on CD8^+^ T cells. As shown in Fig. 2A, the ratio of CD8^+^TIGIT^+^ cells in cervical cancer patients and HSIL patients was significantly higher than that of normal cervix. Additional file 3: Figure S1 shows that the proportion of NK^+^TIGIT^+^ and CD3^+^TIGIT^+^ cells in cervical cancer patients and HSIL patients was significantly higher than in normal cervix. We compared the functions of CD8^+^TIGIT^+^ cells and CD8^+^TIGIT^−^ cells in PBMC. The ability of CD8^+^TIGIT^+^ cells to secrete cytokines (TNF-α and IFN-γ) was significantly lower than that of CD8^+^TIGIT^−^ cells (Fig. 2C), which indicated that CD8^+^TIGIT^+^ cells have low effector function and anti-tumor potential.

Immunohistochemical staining was subsequently performed to investigate TIGIT expression in cervical cancer. Sections of cervical cancer tissues and adjacent cancer tissues were subjected to mIHC. TIGIT and CD8^+^ T cells were labelled for observation under a laser confocal microscopy. We then evaluated the number of CD8^+^TIGIT^+^ cells through mIHC. The number of CD8^+^TIGIT^+^ cells in cervical cancer tissues was significantly higher than that in adjacent cancer tissues (Fig. 2B).

### A database analysis revealed high CD155 expression expressed in cervical cancer patients

**Fig. 3:**
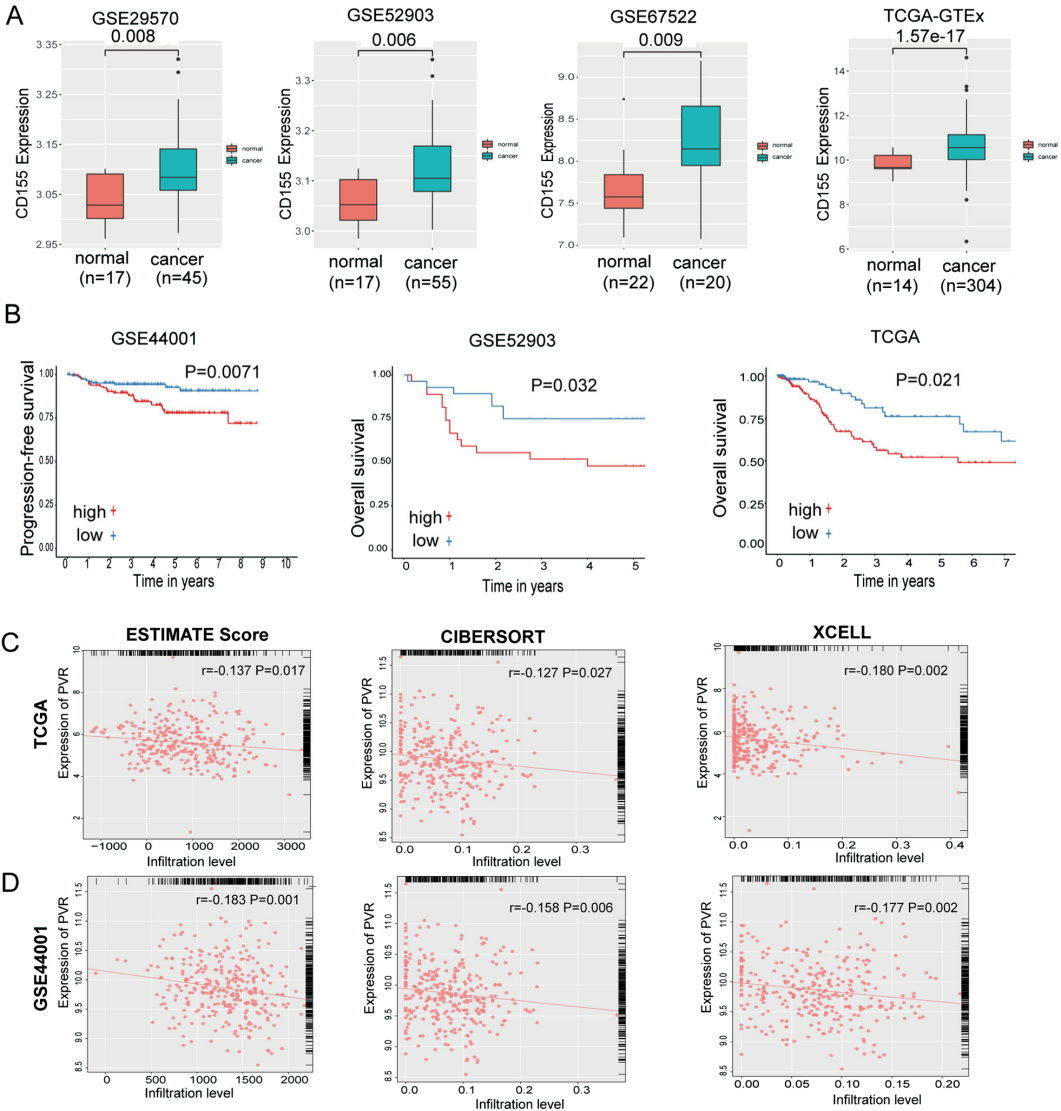
The CD155 expression level in cervical cancer tissues. Correlation analysis between the CD155 expression level and the level of CD8^+^ T cell infiltration. **A** GSE29570, GSE52903, GSE67522 and TCGA-GTEx databases were used to analyse the expression level of the CD155 mRNA in cervical cancer tissues. **B** Results of the Kaplan–Meier survival analysis of the GSE44001, GSE52903, and TCGA databases (GSE44001: n = 300; GSE52903: n = 55; TCGA: n = 304) (**C**) Correlation analysis between the CD155 expression level and the level of CD8^+^ T cell infiltration in TCGA and GSE44001 datasets with the ESTIMATE score, CIBERSORT, and XCELL algorithm. The data are the mean ± SEM of the experiments

CD155 is a high-affinity ligand for TIGIT [10]. The CD155 expression levels in cervical cancer and normal cervical tissue were downloaded from TCGA and GEO databases, the “limma package” was used to analyse differential gene expression, and the data were standardized. In the GSE29570, GSE52903, GSE67522 and TCGA-GTEx datasets, the expression level of CD155 in cervical cancer tissues was higher than that in normal cervical tissues (Fig. 3A, B). We conducted a survival analysis of patients in databases containing survival information, and the results showed that in the GSE44001, GSE52903 and TCGA-GTEx databases, patients with cervical cancer presenting high expression of CD155 experienced shorter progression-free survival or overall survival. Our previous studies confirmed that CD155 expression was elevated in the tissues and plasma of patients with cervical cancer [19].

Next, we used the algorithm to study the cervical cancer database with a large sample size. In the GSE44001 and TCGA datasets, CD155 expression was negatively correlated with the level of CD8^+^ T cells (Fig. 3C, D).

### ***TIGIT/CD155 blockade reverses the inhibitory effect of cervical cancer cells on cytokine production by CD8***^+^***T cells***

**Fig. 4:**
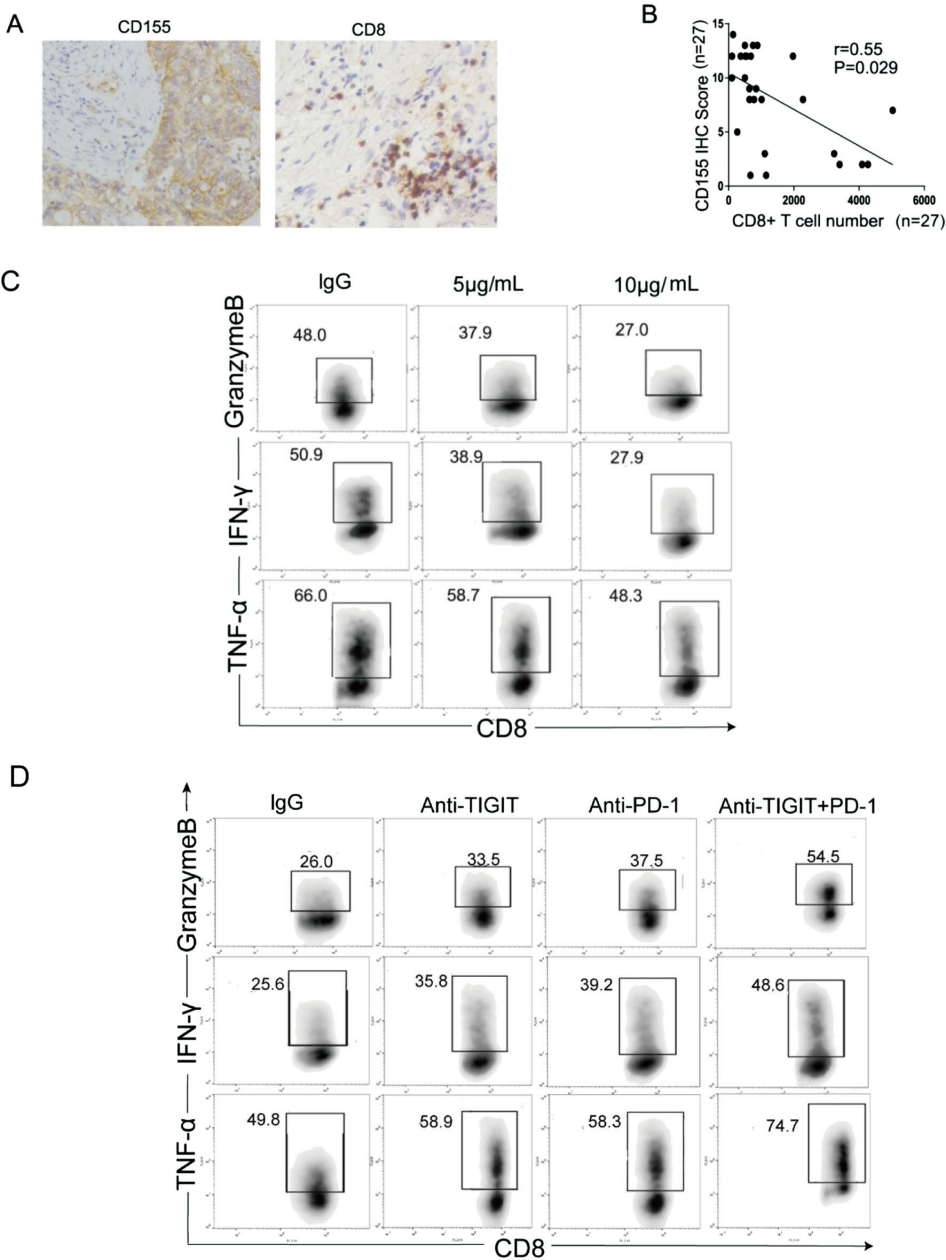
**A**, **B** The CD155 expression level in patients with cervical cancer was related to CD8^+^ T cell depletion. **C** Activated CD8^+^ T cells were treated with CD155-Fc. The ability of CD8^+^ T cells to secrete the cytokines IFN-γ, TNF-α and GranzymeB. **D** CD8^+^ T cells were stimulated with αCD3/CD28 and cocultured with tumour cells in the presence of an anti-TIGIT antibody, anti-PD-1 blocking antibody or isotype control. The production of IFN-γ, TNF-α and GranzymeB in CD8^+^ T cells was detected. The data are at least three independent experiments

The immunohistochemical analysis showed that CD155 was expressed in cervical cancer and negatively correlated with CD8^+^ T cells (Fig. 4A, B). This indicated that the high expression of CD155 inhibited the infiltration of CD8^+^ T cells. We investigated whether CD155 was involved in inhibiting CD8^+^ T cell function. CD8^+^ T cells were sorted from the PBMCs of patients with cervical cancer. Activated CD8^+^ T cells were treated with 5 μg/ml CD155-Fc, 10 μg/ml CD155-Fc. CD8^+^ T cells were collected after 24 h to determine cytokine production using the T cell function assay. Treatment with a high concentration of Fc-CD155 decreased the ability of CD8^+^ T cells to secrete the cytokines IFN-γ, TNF-α and GranzymeB (Fig. 4C). The quantitative analysis is shown in Additional file 4: Figure S2A. CD155 interacted with CD8^+^ T cells and altered CD8^+^ T cell function through CD155/TIGIT.

TIGIT is associated with T cell depletion. We next investigated whether TIGIT blockade modulated the effector function of T cells. The TIGIT blocking antibody was used to block TIGIT signalling. CD8^+^ T cells were stimulated with αCD3/CD28 and cocultured with tumour cells in the presence of an anti-TIGIT blocking antibody or isotype control. Our results showed that the inhibition of of IFN-γ, TNF-α and GranzymeB by CD8^+^ T cells was reversed after blocking TIGIT (Fig. 4D). A study showed that exhausted CD8^+^ T cells coexpress TIGIT and PD-1 [17]. We also investigated whether TIGIT and PD-1 synergistically alter T cell effector functions. Blocking TIGIT or PD-1 increased the production of IFN-γ, TNF-α and GranzymeB in CD8^+^ T cells. Blocking both TIGIT and PD-1 further increased IFN-γ, TNF-α and GranzymeB production in CD8^+^ T cells (Fig. 4D). The quantitative analysis iss shown in Additional file 4: Figure S2B. Based on these results, TIGIT and PD-1 work together to induce CD8^+^ T cell exhaustion.

### TIGIT/CD155 signalling inhibited the activation of the NF-κB and ERK pathways

**Fig. 5:**
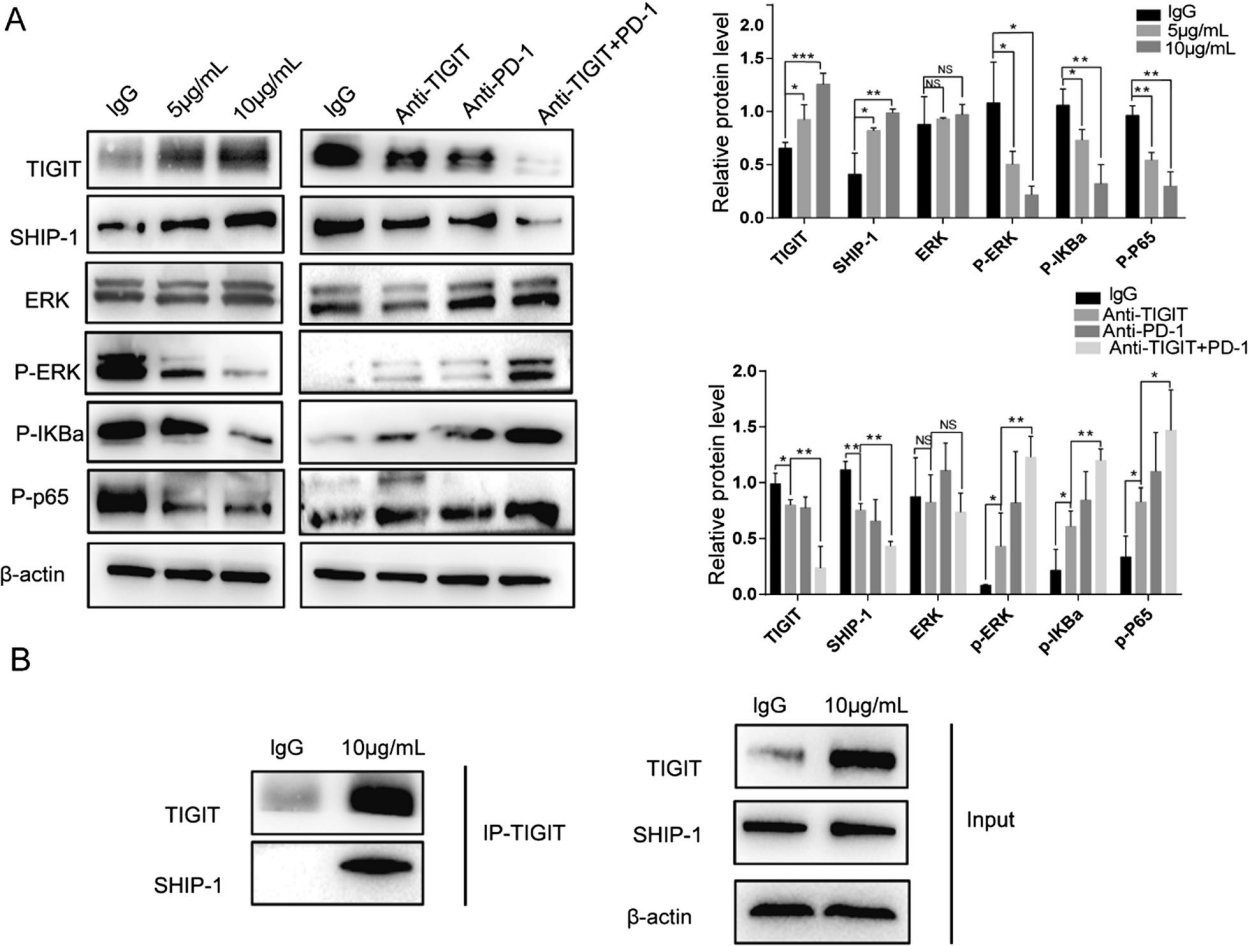
TIGIT/CD155 signalling suppresses the activation of the NF-κB and ERK pathways. **A** Activated CD8^+^ T cells were stimulated with αCD3/CD28 and cocultured with tumour cells in the presence of an anti-TIGIT blocking antibody, anti-PD-1 blocking antibody. Activated CD8^+^ T cells were treated with CD155-Fc. Expression levels of p-ERK/ERK, p-IκBα and p-NF-κBP65. **B** SHIP-1 was associated with negative regulation of T cell activation. We performed Co-IP analysis and observed that TIGIT could precipitate SHIP-1 under the action of Fc-CD155. The data are the mean ± SEM of at least three independent experiments. *P < 0.05, **P < 0.01

NF-κB is a critical transcription factor that controls the production of various cytokines in various immune cells [20]. Previous studies have shown that the mitogen-activated protein kinase (MAPK) and nuclear factor-κB (NF-κB) signalling pathways play an essential role in TIGIT/CD155-mediated immunosuppressive effects on NK cells [21]. Next, we analysed the changes in related proteins in CD8^+^ T cells after an incubation with anti-TIGIT mAb and Fc-CD155. Blocking TIGIT during the culture of activated T cells significantly increased the p-ERK/ERK ratio and p-IκBα and p-NF-κBP65 levels. In addition, the p-ERK/ERK ratio and p-IκBα and p-NF-κBP65 levels were significantly lower in CD8^+^ T cells cultured with Fc-CD155 (Fig. 5A). SHIP-1 is associated with the negative regulation of T cell activation [21]. Early experiments have shown that the engagement of CD3 or CD28 on T cells leads to the phosphorylation and catalytic activation of SHIP-1, indicating a role for SHIP-1 in lymphocyte activation [22]. Silencing SHIP-1 in CD4^+^ T lymphocytes leads to a decrease in the level of the p-ERK protein and reduces the proliferation and motility of CD4^+^ T cells [23]. In our study, blocking TIGIT during the culture of activated CD8^+^ T cells reduced SHIP-1 expression. Blocking both TIGIT and PD-1 further decreased SHIP-1 levels. In activated CD8^+^ T cells cultured with Fc-CD155, SHIP expression was significantly increased.

We performed an immunoprecipitation analysis using CD8^+^ T cells cultured with Fc-CD155 to explore the potential mechanism by which TIGIT/CD155 inhibits the function of CD8^+^ T cells. We performed a coimmunoprecipitation (Co-IP) analysis and observed that TIGIT precipitated SHIP-1 after treatment with Fc-CD155 (Fig. 5B).

### *Targeting TIGIT/CD155 to inhibit tumour progression *in vivo

**Fig. 6:**
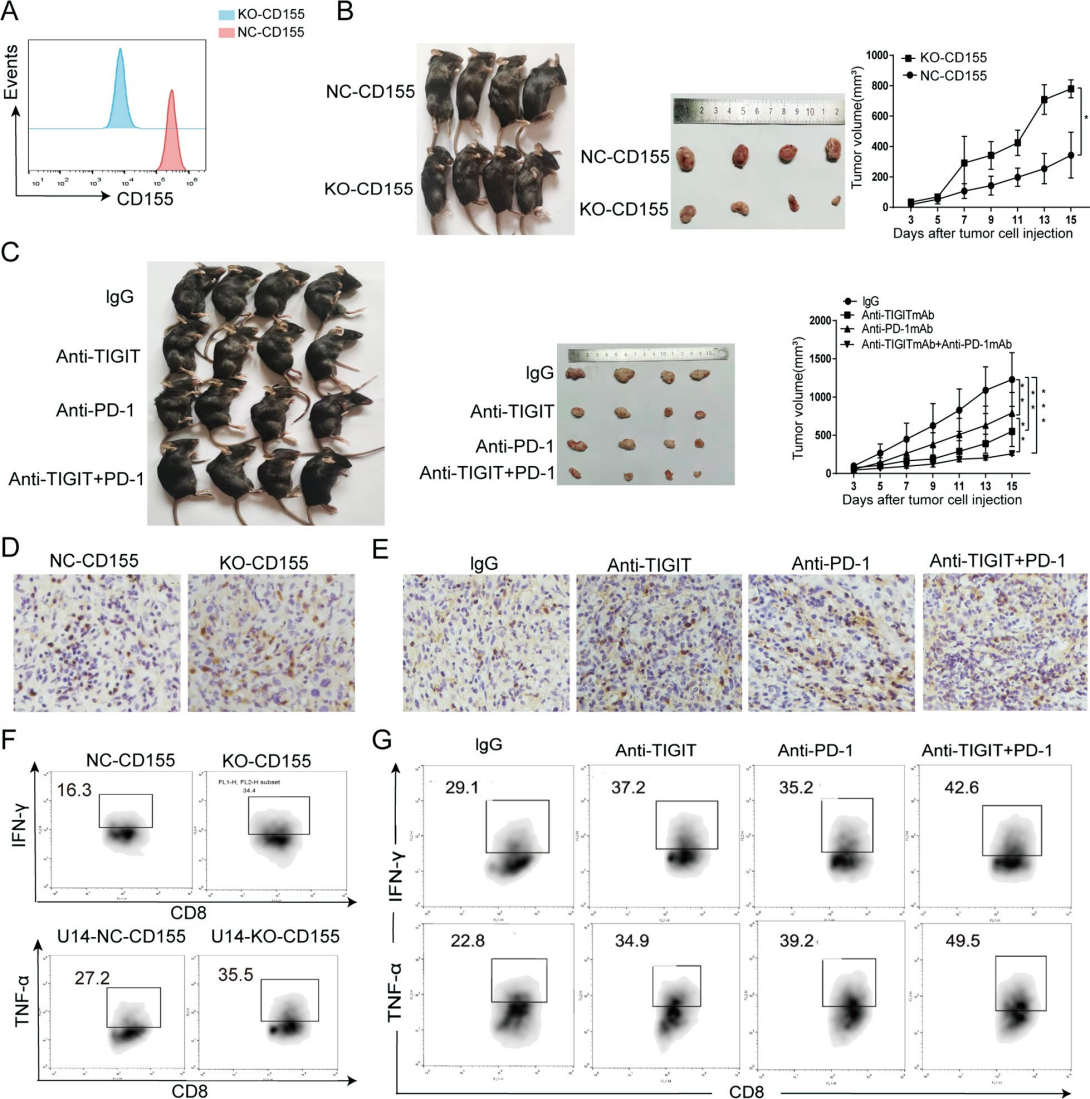
TIGIT/CD155 inhibited the growth of transplanted cervical tumours and the function of CD8^+^ T lymphocytes in C57BL/6 mice. **A** The CD155 knockout efficiency in U14 cells compared with that of the control group. **B** Analysis of tumour progression in mice that were injected with U14-NC-CD155 and U14-KO-CD155 cells. **C** WT-U14 cell-transplanted mice were treated with PD-1 blocking antibodies, TIGIT blocking antibodies or the isotype control. Analysis of tumour progression in mice that received different treatments. **D** IHC analysis of CD8^+^ T cell infiltration level in tumors in mice received U14-NC-CD155 and U14-KO-CD155 cells. **E** IHC analysis of CD8^+^ T cell infiltration levels in tumours from mice that received different treatments. **F** CD8^+^ T cells were isolated from mouse tumour tissues. The levels of TNF-α and IFN-γ secreted by CD8^+^ T cells in mice that received U14-NC-CD155 and U14-KO-CD155 cells were measured. **G** The levels of TNF-α and IFN-γ secreted by CD8^+^ T cells in mice that received different treatments were measured

We constructed tumour models by injecting C57BL6 mice with WT (wild-type) U14, U14-NC-CD155 or U14-KO-CD155 cells to further verify the antitumor effects of targeting TIGIT/CD155 signalling in vivo. Compared with the control group, the CD155 silencing efficiency in U14 cells is nearly 99% (Fig. 6A). Mice injected with WT-U14 cells were treated with PD-1 blocking antibodies, TIGIT blocking antibodies or the isotype control.

Tumour progression was inhibited in mice that received U14- KO-CD155 cells (Fig. 6B). The level of infiltrating CD8^+^ T lymphocytes in the tumour tissue and secretion of TNF-α and IFN-γ levels increased (Fig. 6C, F). The quantitative analysis is shown in Additional file 5: Figure. S3A, C, D.

In WT-U14 injected C57BL/6 mice, the blocking TIGIT inhibited the growth and proliferation of cervical tumours (Fig. 6D). The combined blockade of TIGIT and PD-1 further inhibited tumour growth in C57BL/6 mice compared with the blockade of TIGIT alone. In addition, the combined blockade of TIGIT and PD-1 further increased the level of infiltrating CD8^+^ T lymphocytes in the tumour tissue and secretion function factors TNF-α and IFN-γ by CD8^+^ T lymphocytes compared with the group subjected to TIGIT blockade alone (Fig. 6E, G). The quantitative analysis is shown in Additional file 5: Figure S3B, E, F.

## Discussion

Immunotherapy has joined the ranks of surgery, radiotherapy, chemotherapy, and targeted therapy and has become a cancer treatment modality [24]. Immune escape is a defect in the immune system that promotes carcinogenesis. When naive T cells are activated and differentiate into effector T cells within 1–2 weeks, they acquire (Additional file 1: Figure S1) robust proliferation, transcription, epigenetic, metabolic reprogramming, and effector T cell characteristics. In this developmental process, the gradual loss of effector functions, the continuous upregulation and coexpression of multiple inhibitory receptors, and the expression of key transcription factors all affect the differentiation state of T lymphocytes and lead to T cell depletion [25]. T cell exhaustion is promoted by the activation of inhibitory immune checkpoints (such as PD-1 and CTLA-4) [20]. TIGIT/CD155 has attracted widespread attention as a new immune checkpoint and a potential target for cancer immunotherapy. TIGIT was first identified as an inhibitory receptor that exerts immunosuppressive effects through activated CD4^+^T cells, Tregs and NK cells [10, 21, 22]. Studies examining the immunosuppressive effect of TIGIT on CD8^+^ T cells are increasing. The role and mechanism of TIGIT/CD155 in cervical cancer have not been studied. Our study showed that TIGIT was upregulated in CD8^+^ T cells that infiltrated the tumour tissue of patients with cervical cancer. Compared with cervical tissues, the number of CD8^+^TIGIT^+^ cells in PBMCs and cervical cancer tissues increased significantly. CD155 is the ligand of TIGIT and interacts with TIGIT with high affinity [10]. Loss of host and tumour-derived CD155 expression reduces tumour growth and metastasis and increases the response to immunotherapy [17, 23, 26]. An analysis of TCGA and GEO databases showed higher CD155 expression in cervical cancer tumour tissue than that in normal tissue, and it was related to the shorter survival of patients with cervical cancer. Our previous studies were consistent with the finding that CD155 is expressed at high levels in the plasma and tissues of patients with cervical cancer and is related to cervical cancer progression [19]. In our studies, TIGIT blockade increased cytokines secretion. However, TIGIT levels were significantly upregulated on CD8^+^T cells cultured with Fc-CD155. The activation of TIGIT/CD155 signalling inhibited the immune function of CD8^+^T cells.

TIGIT contains ITT-like phosphorylation motifs and noncanonical ITIM motifs in the cytoplasmic tail [27]. Receptors containing inhibitory ITIMs usually undergo tyrosine phosphorylation of the cytoplasmic tail in the first step of negative signal transduction [28]. Then, they recruit SH2 domain containing tyrosine phosphatases (such as SHP1 and SHP2) or inositol phosphatases (such as SHIP-1 and SHIP-2) to induce inhibitory signalling [29]. Studies assessing the effect of SHIP on macrophage phagocytosis have shown that SHIP expression downregulates Rac activity and subsequent superoxide production [30]. TIGIT-mediated recruitment of SHIP-1 inhibits the NK cell pathway by blocking PI3K and MAPK signalling [31]. The NF-κB protein can regulates the expression of hundreds of genes and important physiological processes such as inflammation, immunity, proliferation, and cell death [32]. The NF-κB pathway regulates the development of T cells and activates TCR signals to trigger T cell functions [33]. We showed that phosphorylated TIGIT binds to CD155 to inhibit NF-κB and ERK activation by recruiting SHIP-1, leading to decreased cytokine production. After blocking TIGIT in inactivated CD8^+^ T cells, the inhibitory effect of SHIP-1 on CD8^+^ T cells was weakened, and the activation of NF-κB and ERK increased.

**Fig. 7:**
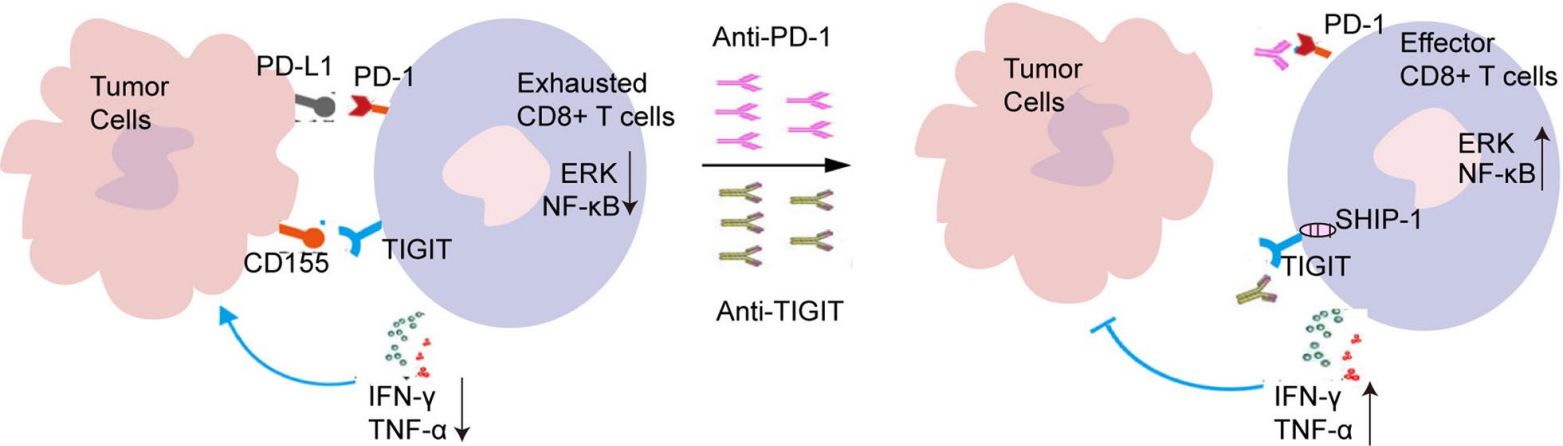
Schematic diagram of CD155/TIGIT binding to regulate CD8^+^ T cell function and related mechanisms

Coexpression of TIGIT and other immune checkpoints might lead to CD8^+^ T cell depletion [17, 18]. Blocking TIGIT and PD-1 increased cytokine production. We confirmed that the combined blockade of TIGIT and PD-1 further enhanced the immune response in tumour-bearing mice and suppressed tumour growth compared with targeting either checkpoint alone (Fig. 7). Thus, a combination of PD-1 and TIGIT inhibitors might represent a useful treatment for cervical cancer.

## Conclusions

In summary, the TIGIT/CD155 signalling exhibits enhanced activity in patients with cervical cancer and mouse models and is related to immunosuppression. Targeting the TIGIT/CD155 signalling pathway may be a potential therapeutic strategy for the treatment of cervical cancer.

## Supplementary Information

**Additional file 1: Table S1.** The primer sequences for the TIGIT, PD-1, LAG3, Tim3 and β-actin.

**Additional file 2: Table S2.** Cervical cancer samples for IHC analysis.

**Additional file 3: Figure S1.** **A** The proportion of NK^+^TIGIT^+^ cells in individuals with a normal cervix, patients with HSILs, and patients with cervical cancer. **B** The proportion of CD3^+^TIGIT^+^ cells in individuals with a normal cervix, patients with HSILs, and patients with cervical cancer. The data are the mean ± SEM of the experiments. *P < 0.05, **P < 0.01, and ***P < 0.001.

**Additional file 4: Figure S2.A** After coculture with CD155-Fc, the percentage of CD8^+^ T cells producing IFN-γ, TNF-α and Granzyme B was analysed. **B** After coculture with the anti-TIGIT mAb or/and anti-PD-1 mAb, the percentage of CD8^+^ T cells producing IFN-γ, TNF-α and Granzyme B was determined. The data are the mean ± SEM of at least three independent experiments. *P < 0.05, **P < 0.01, and ***P < 0.001.

**Additional file 5: Figure S3. A** The level of CD8^+^ T cell infiltration in tumour tissues from U14-CD155-KO and U14-CD155-NC cell-transplanted mice. **B** Level of infiltrating CD8^+^ T cells after an injection of the TIGIT or PD-1 blocking antibodies. **C**, **D** Levels of TNF-α and IFN-γ secreted by infiltrated CD8^+^ T cells in the U14-CD155-KO group and U14-KO-CD155 group. **E**, **F** Levels of TNF-α and IFN-γ secreted by CD8^+^ T lymphocytes treated with the combination of the anti-TIGIT mAb or/and anti-PD-1 mAb. *P < 0.05, **P < 0.01, and ***P < 0.001.

## Data Availability

The data used and/or analysis during the current study are available from the corresponding author on reasonable request.
