# Supplementary material for: Blocking TIGIT/CD155 signalling reverses CD8+ T cell exhaustion and enhances the antitumor activity in cervical cancer
Source: J Transl Med. 2022 Jun 21;20:280. doi: 10.1186/s12967-022-03480-x (PMC9210727; doi:10.1186/s12967-022-03480-x)
Supplement: Supplementary file 1 — Additional file 1: Table S1. The primer sequences for the TIGIT, PD-1, LAG3, Tim3 and β-actin. [file 12967_2022_3480_MOESM1_ESM.docx]

**Additional file 1: Table S1**

The primer sequences for the TIGIT, PD-1, LAG3, Tim3 and β-actin.

|  | Primer sequence |  |
| --- | --- | --- |
| TIGIT-F | 5'- GACTTGGGGTGGCACATCTC -3' | Human |
| TIGIT-R | 5'- CGTCCCATCAGGGTAGGTGT -3' | Human |
| PD-1-F | 5'- AGGATGGTTCTTAGACTCCCCA -3' | Human |
| PD-1-R | 5'- CACGAAGCTCTCCGATGTGT -3' | Human |
| LAG3-F | 5'- AACCTCACTGTTCTGGGTCTG -3' | Human |
| LAG3-R | 5'- AGTCGCCATTGTCTCCAGTC -3' | Human |
| Tim3-F | 5'- TCATCAAACCAGCCAAGGTCA -3' | Human |
| Tim3-R | 5'- GTCCCCTGGTGGTAAGCATC -3' | Human |
