# Supplementary material for: Blocking TIGIT/CD155 signalling reverses CD8+ T cell exhaustion and enhances the antitumor activity in cervical cancer
Source: J Transl Med. 2022 Jun 21;20:280. doi: 10.1186/s12967-022-03480-x (PMC9210727; doi:10.1186/s12967-022-03480-x)
Supplement: Supplementary file 2 — Additional file 2: Table S2. Cervical cancer samples for IHC analysis. [file 12967_2022_3480_MOESM2_ESM.docx]

**Additional file 2: Table S2.**

Cervical cancer samples for IHC analysis.

|  | positive cells | | Positive cells/mm^3^ | |  | Positive cells | | Positive cells/mm^3^ |
| --- | --- | --- | --- | --- | --- | --- | --- | --- |
| Cancer 1 | | 219 | 1586 | Adjacent cancer 1 | | | 7 | 47 |
| Cancer 2 | | 125 | 906 | Adjacent cancer 2 | | | 19 | 135 |
| Cancer 3 | | 1156 | 8187 | Adjacent cancer 3 | | | 65 | 610 |
| Cancer 4 | | 153 | 1086 | Adjacent cancer 4 | | | 19 | 118 |
| Cancer 5 | | 512 | 3624 | Adjacent cancer 5 | | | 52 | 515 |
| Cancer 6 | | 349 | 2494 | Adjacent cancer 6 | | | 162 | 1150 |
| Cancer 7 | | 84 | 593 | Adjacent cancer 7 | | | 138 | 986 |
| Cancer 8 | | 598 | 4237 | Adjacent cancer 8 | | | 293 | 2928 |
| Cancer 9 | | 657 | 4651 | Adjacent cancer 9 | | | 150 | 1062 |
| Cancer 10 | | 416 | 3050 | Adjacent cancer 10 | | | 7 | 47 |
| Cancer 11 | | 1196 | 8528 | Adjacent cancer 11 | | | 18 | 145 |
