# Supplementary figures and images for: Blocking TIGIT/CD155 signalling reverses CD8+ T cell exhaustion and enhances the antitumor activity in cervical cancer
Source: J Transl Med. 2022 Jun 21;20:280. doi: 10.1186/s12967-022-03480-x (PMC9210727; doi:10.1186/s12967-022-03480-x)

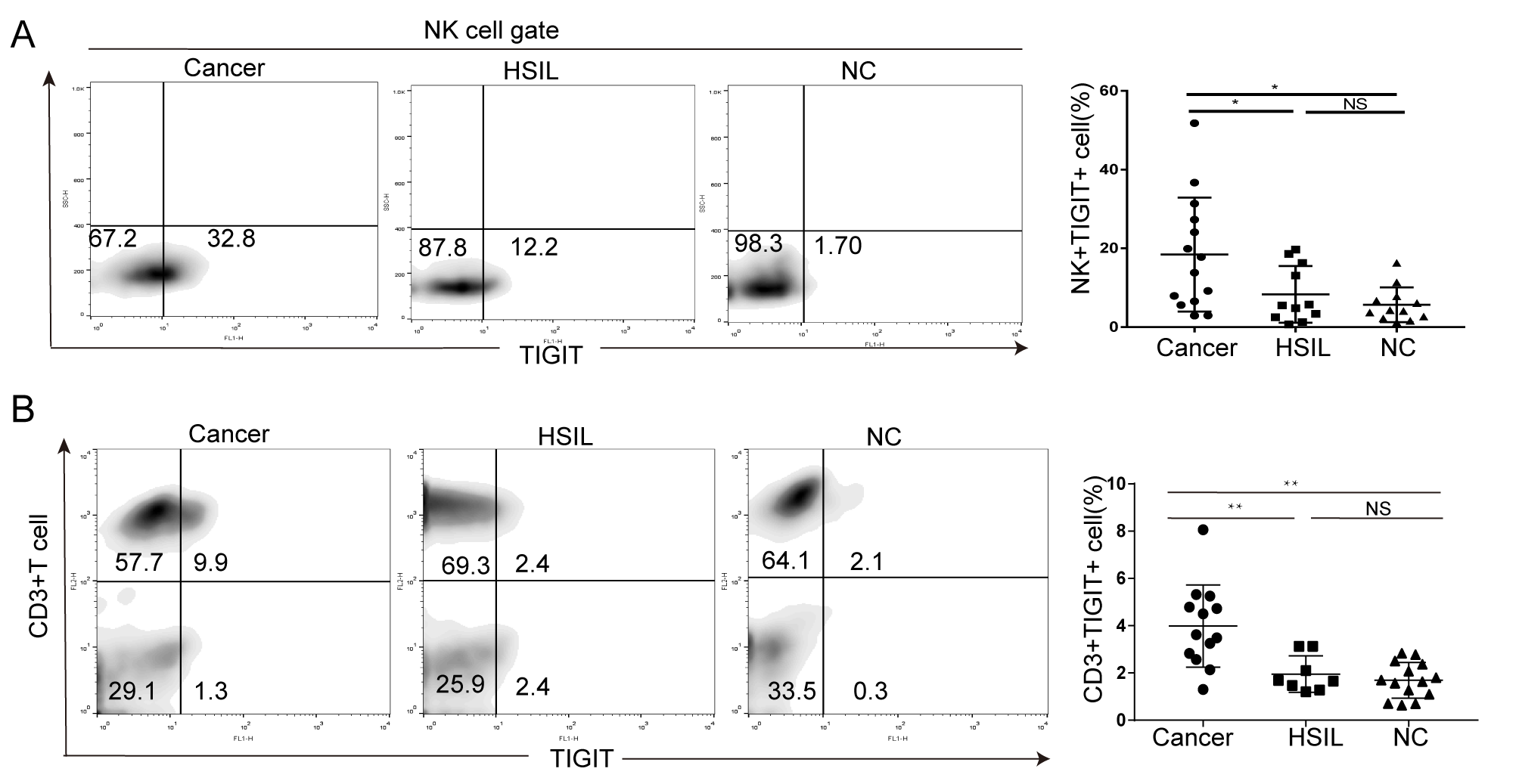

Supplement: Supplementary file 3 — Additional file 3: Figure S1. A The proportion of NK+TIGIT+ cells in individuals with a normal cervix, patients with HSILs, and patients with cervical cancer. B The proportion of CD3+TIGIT+ cells in individuals with a normal cervix, patients with HSILs, and patients with cervical cancer. The data are the mean ± SEM of the experiments. *P < 0.05, **P < 0.01, and ***P < 0.001. [file 12967_2022_3480_MOESM3_ESM.tif]

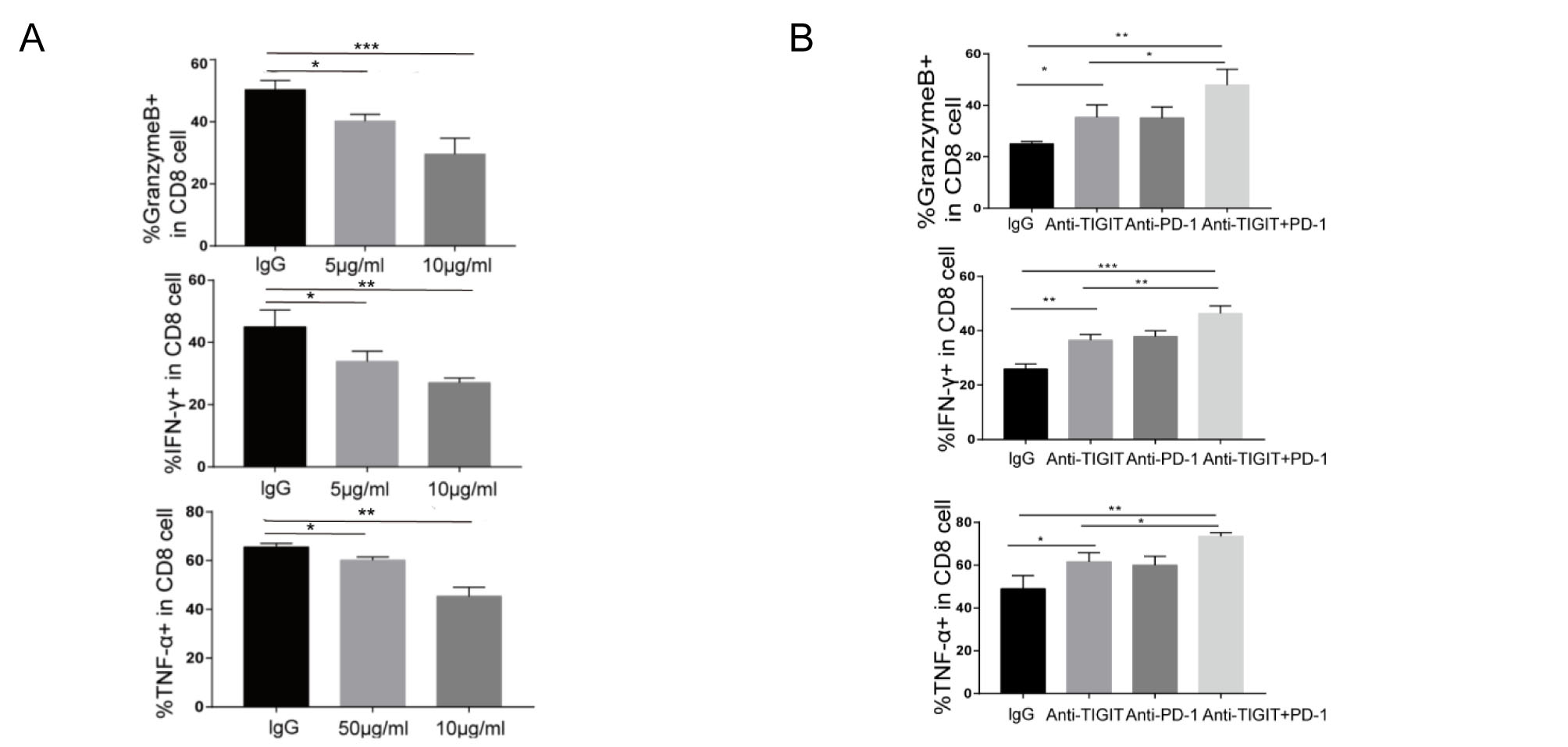

Supplement: Supplementary file 4 — Additional file 4: Figure S2.A After coculture with CD155-Fc, the percentage of CD8+ T cells producing IFN-γ, TNF-α and Granzyme B was analysed. B After coculture with the anti-TIGIT mAb or/and anti-PD-1 mAb, the percentage of CD8+ T cells producing IFN-γ, TNF-α and Granzyme B was determined. The data are the mean ± SEM of at least three independent experiments. *P < 0.05, **P < 0.01, and ***P < 0.001. [file 12967_2022_3480_MOESM4_ESM.jpg]

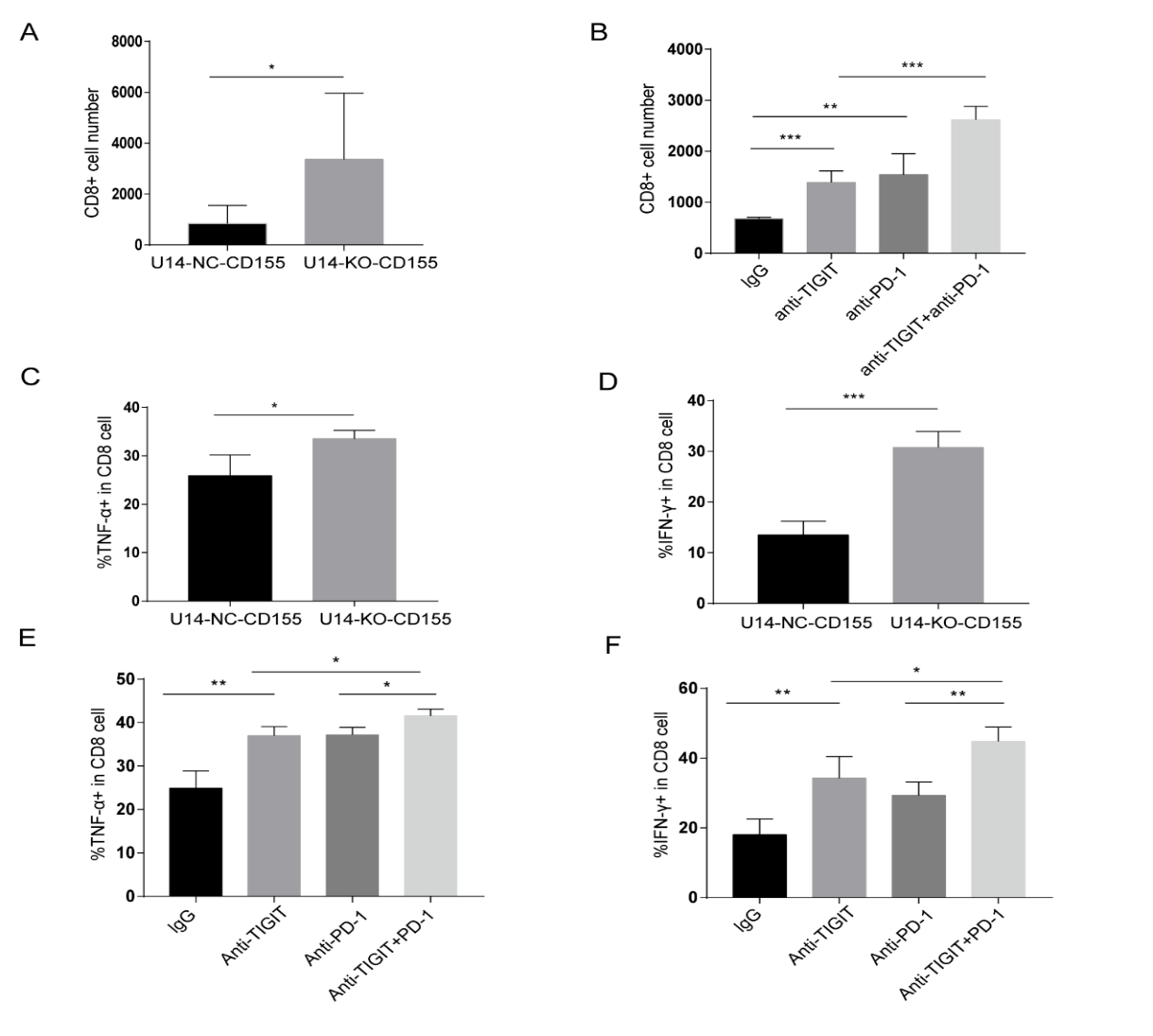

Supplement: Supplementary file 5 — Additional file 5: Figure S3. A The level of CD8+ T cell infiltration in tumour tissues from U14-CD155-KO and U14-CD155-NC cell-transplanted mice. B Level of infiltrating CD8+ T cells after an injection of the TIGIT or PD-1 blocking antibodies. C, D Levels of TNF-α and IFN-γ secreted by infiltrated CD8+ T cells in the U14-CD155-KO group and U14-KO-CD155 group. E, F Levels of TNF-α and IFN-γ secreted by CD8+ T lymphocytes treated with the combination of the anti-TIGIT mAb or/and anti-PD-1 mAb. *P < 0.05, **P < 0.01, and ***P < 0.001. [file 12967_2022_3480_MOESM5_ESM.tif]
